# Monitoring within-country health inequalities:  the example of Indonesia

**DOI:** 10.1080/16549716.2018.1545626

**Published:** 2018-11-26

**Authors:** Lubna Al-Ansary, Navaratnasamy Paranietharan, Siswanto

**Affiliations:** a Assistant Director General, Health Metrics and Measurement Cluster, World Health Organization, Geneva, Switzerland; b WHO Representative, Country Office for Indonesia, World Health Organization, Jakarta, Indonesia; c Director General, National Institute of Health Research and Development, Ministry of Health, Jakarta, Indonesia

The world is united on the path to achieving the United Nations’ Sustainable Development Goals (SDGs) – in which, specifically in the realm of health, Universal Health Coverage is the central strategy for ‘leaving no-one behind.’ Fulfilling this commitment requires vigilance and political will on the part of each country through health inequality monitoring [1].

As this Special Issue demonstrates, Indonesia is a model of how the health-related SDGs may be achieved by individual countries, in a truly collaborative fashion. Technical support from the World Health Organization (WHO) – from its Geneva headquarters through its regional office to the country office in Jakarta – working in close partnership with the Indonesian Ministry of Health as well as critical stakeholders in the country, has led to this watershed exercise and serious reflection and action on health inequalities in Indonesia. The process was mutually beneficial, in that Indonesia was able to carry out analyses related to inequality while supporting and implementing the development of health inequality monitoring tools under the aegis of WHO. The path for other WHO Member States may look slightly different, but it is already paved by Indonesia’s commitment and example.

WHO’s Thirteenth General Programme of Work calls for strategically disaggregating data when they are collected, analysed and reported across data sources, identifying health inequalities and their drivers to improve programme delivery, and acknowledging the centrality of health information systems for monitoring health inequalities [2]. WHO is committed to extending such support to additional equity-oriented activities that Indonesia may wish to undertake as well as other nations on this path. A range of tools and processes have been put in place in support of this. This includes publications such as the *Handbook on health inequality monitoring with a special focus on low- and middle-income countries* [3], a detailed manual for national health inequality monitoring [4], and a variety of reports that outline the process and demonstrate the output of monitoring the state of inequality at the global and national levels, and in relation to specific domains [5–7]. In addition, tools and resources such as the Health Equity Assessment Toolkit [8,9] and the Health Equity Monitor [10] can provide further guidance and facilitate monitoring.

Achieving the SDGs requires repeated, iterative efforts. This Special Issue demonstrates that health inequality monitoring can be a model for the content and process of such efforts by promoting continuous, mutual learning, and an explicit, operational commitment to equity.
